# Lateralization of FDG-PET Hypometabolism Using Resting-State fMRI in Temporal Lobe Epilepsy: A Simultaneous PET-MRI Study

**DOI:** 10.3390/tomography12030030

**Published:** 2026-03-02

**Authors:** Daniel Uher, Gerhard S. Drenthen, Tineke van de Weijer, Jochem van der Pol, Christianne M. Hoeberigs, Paul A. M. Hofman, Sam Springer, Rob P. W. Rouhl, Albert J. Colon, Olaf E. M. G. Schijns, Walter H. Backes, Jacobus F. A. Jansen

**Affiliations:** 1 Department of Radiology and Nuclear Medicine, Maastricht University Medical Centre, 6229 HX Maastricht, The Netherlands; daniel.uher@ovgu.de (D.U.);; 2 Mental Health and Neuroscience Research Institute (MHeNs), Maastricht University, 6229 ER Maastricht, The Netherlands; 3 Department of Neurosurgery, Maastricht University Medical Center, 6229 HX Maastricht, The Netherlands; 4 Department Biomedical Magnetic Resonance (BMMR), Otto von Guericke University Magdeburg, 39120 Magdeburg, Germany; 5 Institute of Nutrition and Translational Research in Metabolism (NUTRIM), Maastricht University, 6229 ER Maastricht, The Netherlands; 6 Academic Center for Epileptology, Kempenhaeghe and Maastricht University Medical Centre, 5591 VE Heeze, The Netherlands; 7 Care and Public Health Research Institute (CAPHRI), Maastricht University, 6229 ER Maastricht, The Netherlands; 8 Department of Radiology, Horatio Oduber Hospital, Oranjestad, Aruba; 9 Department of Neurology, Maastricht University Medical Center, 6229 HX Maastricht, The Netherlands; 10 Centre d‘Etudes et Traitement de l‘Epilepsie, CHU-Martinique, 97200 Fort-de-France, France; 11 Cardiovascular Research Institute Maastricht (CARIM), Maastricht University, 6229 ER Maastricht, The Netherlands; 12 Department of Electrical Engineering, Eindhoven University of Technology, 5612 AP Eindhoven, The Netherlands

**Keywords:** simultaneous PET-MRI, hybrid PET-MRI, temporal lobe epilepsy, resting-state fMRI, lateralization, fALFF

## Abstract

Background: In temporal lobe epilepsy (TLE), locally reduced glucose metabolism (i.e., hypometabolism) is indicative of the epileptogenic onset zone (EZ). Here, we investigate the potential value of resting-state fMRI (rs-fMRI) for localizing the EZ with fluorodeoxyglucose positron emission tomography (FDG-PET) as ground truth. Methods: Twelve PET-positive patients (34.1 ± 13.1 y; 5 females) with unilateral drug-resistant TLE were included. FDG-PET and rs-fMRI were acquired simultaneously at a hybrid 3T PET-MR scanner. Hypometabolic regions were identified on the FDG-PET images by a nuclear medicine expert. The FDG-PET images were compared with a clinical FDG-PET control dataset with normal glucose uptake distribution. The output z-score maps were thresholded at z < −2 to produce a binary mask of the significantly hypometabolic regions. The hypometabolism masks were mirrored onto the contralateral hemisphere for the asymmetry comparison. Regional homogeneity (ReHo), amplitude of low-frequency fluctuations (ALFF), and fractional ALFF (fALFF) were calculated from the rs-fMRI in conventional (0.01–0.1 Hz) and slow-3 (0.073–0.198 Hz) frequency bands. Asymmetry indices (AIs) were calculated using the ipsilateral and contralateral hypometabolic masks in the PET-positive subjects and assessed via the one-sample Wilcoxon test and Spearman correlation coefficients. Results: The AIs of conventional fALFF were significantly lower in the hypometabolic zone (*p* < 0.05). A significant negative correlation was found between the AIs of FDG-PET and fALFF in the slow-3 band (r = −0.62; *p* < 0.05). Conclusions: Conventional and slow-3 band fALFF showed a potential to mimic the FDG-PET findings in terms of EZ localization. Further research with extended cohorts and histopathological validation is required to determine the clinical value.

## Simple Summary

This study investigated whether resting-state fMRI (rs-fMRI) can localize the epileptogenic onset zone in temporal lobe epilepsy (TLE) using FDG-PET as ground truth. Twelve patients with drug-resistant TLE underwent simultaneous PET-MR scanning at 3T. Hypometabolic regions identified on FDG-PET were compared with rs-fMRI metrics including regional homogeneity (ReHo), amplitude of low-frequency fluctuations (ALFF), and fractional ALFF (fALFF) calculated in conventional and slow-3 frequency bands. Results showed that fALFF asymmetry indices were significantly reduced in hypometabolic zones, with a significant negative correlation between FDG-PET and slow-3 fALFF (r = −0.62, *p* < 0.05). These findings suggest fALFF may replicate FDG-PET findings for epileptogenic zone localization.

## 1. Introduction

Globally, approximately 30% of individuals with drug-resistant focal epilepsy (DRE) continue experiencing seizures despite treatment with antiseizure medication (ASM) [1]. For certain patients, epilepsy surgery represents an evidence-based and curative treatment option [2,3]. When feasible, surgical resection of the epileptogenic zone (EZ) offers a high chance of post-surgical seizure freedom [4]. Detection of a structural and/or functional lesion on MRI is a major predictive preoperative factor for a favorable seizure outcome after surgery [5]. Despite the well-described variety of pathological lesions responsible for anatomical and/or functional alterations within and around the EZ, accurate lateralization and/or even localization remain a substantial challenge [6,7,8,9].

Resting-state blood-oxygen-level-dependent functional MRI (rs-fMRI) represents a potential tool for lateralizing and localizing the EZ. Commonly derived rs-fMRI quantitative metrics are regional homogeneity (ReHo; measure of localized temporal synchronicity amongst neighboring voxels; [10]), amplitude of low-frequency fluctuations (ALFF; the sum of measured amplitudes within a specified frequency bandwidth, most commonly 0.01–0.1 Hz; [11,12]), and fractional ALFF (fALFF; a ratio between the ALFF value and the integral of the measured amplitude spectrum; [12]). Previous studies have reported detectable alterations in these metrics within and around the EZ [13,14,15] and, therefore, highlight the potential diagnostic benefit [16,17]. Additionally, utilizing temporal bandpass filters with wider bandwidths could increase the sensitivity to the epilepsy-induced alterations in the rs-fMRI power spectra [18].

Fluorodeoxyglucose positron emission tomography (FDG-PET) has proven to be a valuable tool for presurgical workup in DRE patients [19,20]. Compared with ictal single- photon emission computed tomography, FDG-PET can be acquired interictally while maintaining strong sensitivity to local glucose-uptake aberrations often found in TLE patients [20,21]. Interictal epileptogenic activity is frequently associated with localized reduced glucose metabolism (hypometabolism), a strong indicator of post-surgical seizure outcome, especially in TLE [19,22,23]. However, FDG-PET remains a costly examination utilizing a radioactive intravenous tracer; therefore, alternative methods that provide similar information to FDG-PET while addressing the current drawbacks are necessary.

The relationship between FDG-PET and rs-fMRI is of significant interest, with studies reporting measurable associations between glucose uptake patterns and quantitative fMRI-derived metrics [21,24]. While the physiological mechanisms of both modalities differ, correlations between fMRI-derived metrics and interictal glucose uptake values have previously been observed in mesial temporal lobe epilepsy patients [10]. However, the precise mechanism of the interplay between the blood-oxygen-level-dependent effect and glucose uptake remains ambiguous in patients with TLE. Establishing that fMRI can provide similar lateralization and localization information as FDG-PET would support its use as a complementary modality in the presurgical workup, thus potentially reducing radiation burden and lowering healthcare costs.

As such, in this proof-of-principle study, we investigated the potential of rs-fMRI for lateralizing and localizing the EZ using simultaneously acquired rs-fMRI and FDG-PET images.

## 2. Materials and Methods

### 2.1. Patient Scanning and Selection

**Figure 1:**
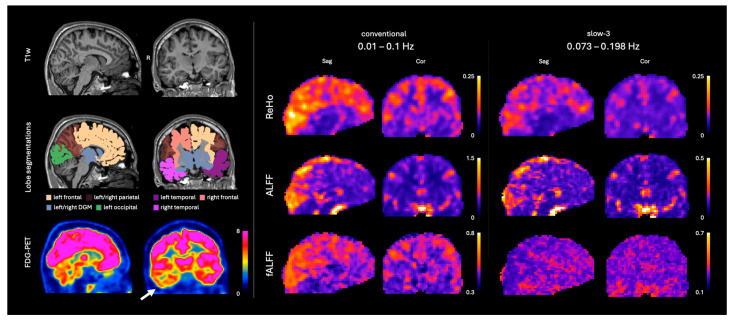
Overview of the utilized images and masks from a representative case (15 y male). The white arrow indicates the right-sided temporal lobe hypometabolism. T1w: T1-weighted image; FDG-PET: fluorodeoxyglucose positron emission tomography; ReHo: regional homogeneity; ALFF: amplitude of low-frequency fluctuations; fALFF: fractional ALFF; DGM: deep grey matter; Sag: sagittal view; Cor: coronal view.

Altogether, 118 patients with DRE who were in the epilepsy presurgical workup trajectory underwent a clinical simultaneous 3T PET-MRI scan (Siemens Biograph mMR, Siemens Healthcare) with the following sequences: T1-weighted (T1w) magnetization prepared rapid gradient echo (MPRAGE); rs-fMRI; and static FDG-PET. Image examples are shown in Figure 1, and relevant sequence parameters in Table 1. The patients were to refrain from any physical activity 1 day prior to the scan. The FDG dose was adjusted for each individual according to their body weight. After tracer injection, patients rested on a bed in a room with dimmed light and were not allowed to sleep then or during the scan. All FDG-PET images were clinically evaluated by an epilepsy-trained nuclear radiologist, and all MRI images were evaluated by an experienced neuroradiologist. All patients signed an informed consent form before scanning. From the full scanned cohort, patients were filtered according to the following criteria: (1) >12 years of age, (2) temporal lobe onset according to electroencephalography (EEG) evaluation, (3) rs-fMRI scan including a reverse phase-encoding scan for post-processing echo planar imaging distortion correction, and (4) positive for unilateral hypometabolic findings in the FDG-PET images in a clinical assessment by a nuclear medicine expert, i.e., PET-positive.

As a result, 12 PET-positive patients—34.1 ± 13.1 y (mean ± SD); 5 females—were included in this study. The FDG dose distribution was 1.56 ± 0.13 MBq/kg (mean ± SD). Furthermore, 6/12 underwent stereo-EEG and 9/12 underwent a magnetoencephalography assessment. All 12 included patients demonstrated unilateral temporal lobe hypometabolism on FDG-PET concordant with temporal lobe seizure onset determined by scalp EEG evaluation, ensuring multimodal concordance for EZ lateralization. The final cohort demographics can be found in Table 2.

### 2.2. FDG-PET Analysis

The raw FDG-PET images were converted to standard uptake values (SUV) (accounting for body weight, dose, nuclide half-time, and injection-to-scan delay) using

(1) $${SUV}_{x,y,z}=\frac{{PET}_{x,y,z}}{\frac{TD}{BW}}\left[g/mL\right]$$

for every voxel position _(x,y,z)_, where PET_x,y,z_ is the measured 18F-FDG activity concentration in the voxel, BW is the patient body weight, and TD is the decay-corrected total injected 18F-FDG dose. The SUV conversion normalizes the FDG-PET signal for injected dose and body weight, thus creating a quantitative metric applicable across the cohort.

### 2.3. Resting-State fMRI Processing

The acquired rs-fMRI datasets were topup corrected for geometric distortions [25] using FSL [26]. Subsequently, the data was corrected for differences in slice timing, realigned, and motion-corrected using the 24 standard motion parameters and the RESTplus toolbox v1.3 [27] based in MATLAB R2023b (MathWorks, Natick, MA, USA). All 175 volumes in each dataset were kept, and no scrubbing was performed. The data was further spatially smoothed using a Gaussian kernel with FWHM = 4 mm. Consequently, the time series was filtered using two bandpass filters separately. In addition to the conventional band (0.01–0.1 Hz), we analyzed the slow-3 band (0.073–0.198 Hz) based on a frequency decomposition scheme proposed by Wang et al. (2014) [28] for epilepsy research and further researched by Gupta et al. (2018) [18]. Both demonstrate that finer frequency sub-bands may reveal BOLD abnormalities relevant to the epileptic condition. Thus, the inclusion of both bands allows the comprehensive characterization of the BOLD signal, as different frequency ranges may reflect distinct physiological processes and pathological mechanisms in epileptogenic tissue. Thus, two separate rs-fMRI datasets were created, each retaining different frequency ranges: the conventional band (0.01–0.1 Hz) and, additionally, the so-called slow-3 band (0.073–0.198 Hz), previously highlighted as a potential bandwidth sensitive to epileptic abnormalities mainly in ALFF [18].

**Figure A1:**
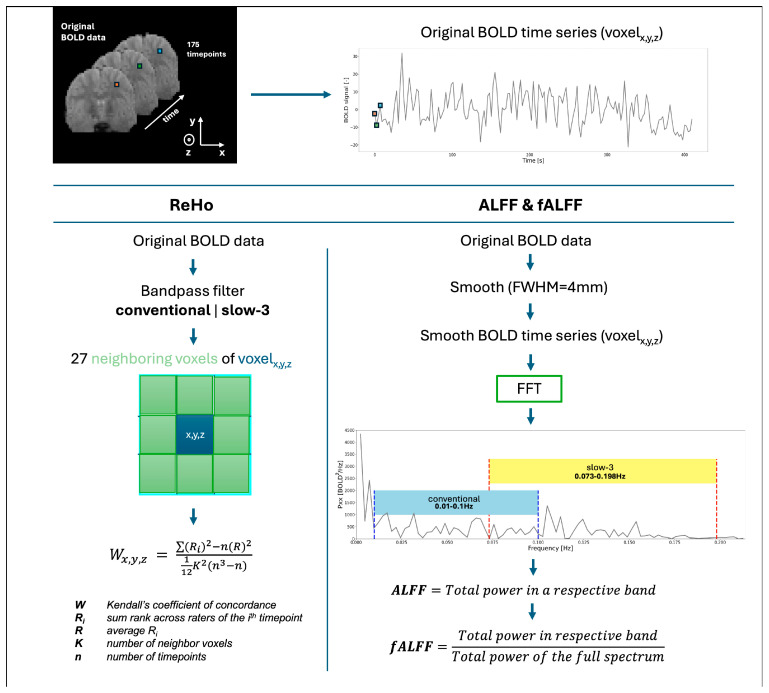
Visual explanation of ReHo, ALFF, and fALFF calculations with the conventional and slow-3 frequency bands. The visualization is rendered as two-dimensional; however, all image operations are done in three dimensions. The “slow-3” mark refers to the frequency 0.073–0.193 Hz, while the “conventional” mark denotes the 0.01–0.1 Hz band. BOLD: blood-oxygen-level-dependent; ReHo: regional homogeneity; ALFF: amplitude of low-frequency fluctuations; fALFF: fractional ALFF; FFT: Fast Fourier Transform; FWHM: full width half maximum.

Regional homogeneity (ReHo) was calculated using Kendall’s coefficient of concordance [10]. The ReHo map was calculated from the unsmoothed data and smoothed after the calculation using the same kernel, to avoid artificially boosting the ReHo values. Both ALFF and fALFF were calculated from the spatially smoothed data without bandpass filtering to diminish artificial noise artifacts. All rs-fMRI metrics were calculated for the two separate frequency bands: (1) conventional (0.01–0.1 Hz), named ‘ReHo’, ‘ALFF’ and ‘fALFF’; and (2) slow-3 (0.073–0.198 Hz), named ‘ReHo slow-3’, ‘ALFF slow-3’ and ‘fALFF slow-3’. Detailed visualization of the calculation of each rs-fMRI metric can be found in Appendix A Figure A1.

Due to the constraints within a clinical setting, fMRI acquisition was not optimal. The field of view of the fMRI was missing the inferior (anterior) part of the temporal lobe and suffered from signal dropouts in the inferior posterior temporal lobe areas due to the proximal air cavities. Therefore, the inferior temporal lobe cortex (as segmented by Freesurfer [29]) was excluded from the analysis.

Furthermore, the two bottom slices of the metric maps were excluded due to ReHo’s propagation of the edge zeros through the kernel and ‘dimming’ the bottom two slices. The excluded areas partially overlap with the hypometabolisms in some of the subjects and therefore were removed to avoid false-positive findings.

### 2.4. Contralateral Asymmetry Analysis

The PET-positive patient images were clinically evaluated in the MIMVista v7.4.7 software application (MIM Software, Cleveland, OH, USA) by epilepsy-trained nuclear radiologists. The data was processed via a standard processing pipeline: (1) The PET images and the T1w images were coregistered to a symmetrical template (further called the ‘MIMVista space’); (2) The PET images were voxel-wise compared against a completely separate clinical PET control database (43 subjects, 63.8 ± 9.9 y; 19 females) that is often utilized by nuclear radiologists during evaluations; (3) The comparison resulted in a z-score map, where negative values indicate hypometabolism with respect to the control database; and (4) The z-score map was thresholded at −2 and binarized to produce a mask (further called ‘the z-score mask’) of the hypometabolic regions indicative of the symptomatic zone related to the epileptic condition. The resulting masks were extracted from the software and used for further processing.

The ReHo, ALFF, and fALFF masks were spatially coregistered using mri_coreg, mri_vol2vol, and antsRegistrationSyNQuick.sh [29,30] to the MIMVista space, using the T1w for the registration parameter estimation. The binary hypometabolism masks were mirrored onto the contralateral hemisphere (left-to-right or right-to-left) to produce the contralateral mask. Asymmetry index (AI) was calculated between the ipsi- and contra- lateral masks as

AI = (ipsilateral − contralateral)/(ipsilateral + contralateral)(2)

where ipsilateral and contralateral are the median values of the given metric from the corresponding masks. Ipsilateral refers to the hypometabolic side while contralateral to the non-hypometabolic side. All analysis steps outside of MIMVista were done in Python v3.10 [31,32].

### 2.5. Statistical Analysis

Outliers in the asymmetry indices across all reconstructed quantitative metrics were identified using Cook’s distance [33], with Cook’s distance of 1.0 considered the threshold for outliers. To evaluate the lateralization performance, asymmetry indices were tested against zero using a one-sample Wilcoxon signed-rank test (*p* < 0.05 for significance). Additionally, Spearman correlation coefficients were computed between the asymmetry indices across subjects to evaluate potential associations between the quantitative metrics. Correlation coefficients with an absolute value over 0.5 were considered strong [34].

## 3. Results

Cook’s distances remained below 1 across all metrics for every subject, indicating the absence of influential outliers. Two subjects exhibited elevated Cook’s distances of 0.53 and 0.33 for fALFF slow-3. A detailed visual inspection of these cases revealed no evidence of artifacts.

**Figure 2:**
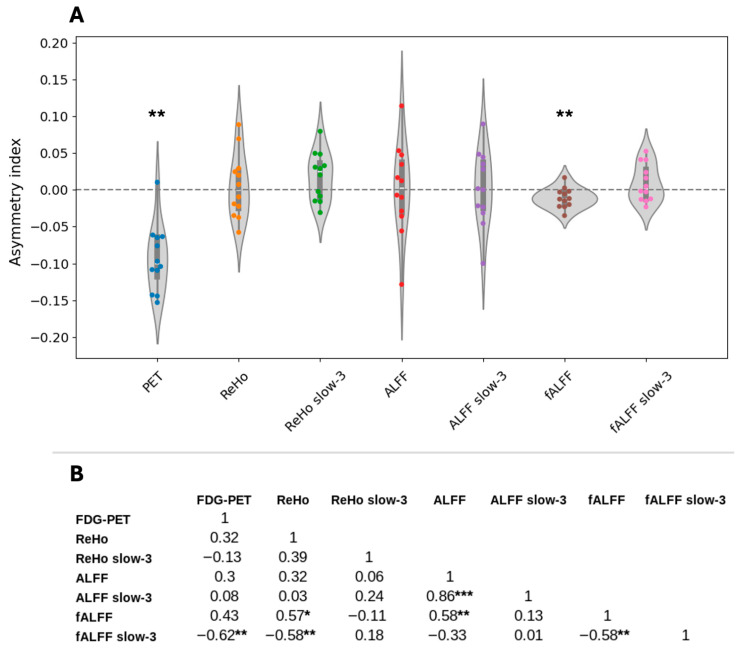
(**A**) Asymmetry indices of all metrics; (**B**) Spearman correlation coefficients between the respective indices. The asterisk denotes the correlation significance as follows: * for *p* < 0.1; ** for *p* < 0.05; *** for *p* < 0.01. For both A and B, ReHo, ALFF, and fALFF refer to the metrics derived from the conventional frequency band of 0.01–0.1 Hz. The metrics with the “slow-3” mark refer to the frequency band 0.073–0.198 Hz. ALFF: amplitude of low-frequency fluctuations; fALFF: fractional ALFF; FDG-PET: fluorodeoxyglucose positron emission tomography; ReHo: regional homogeneity.

**Figure 3:**
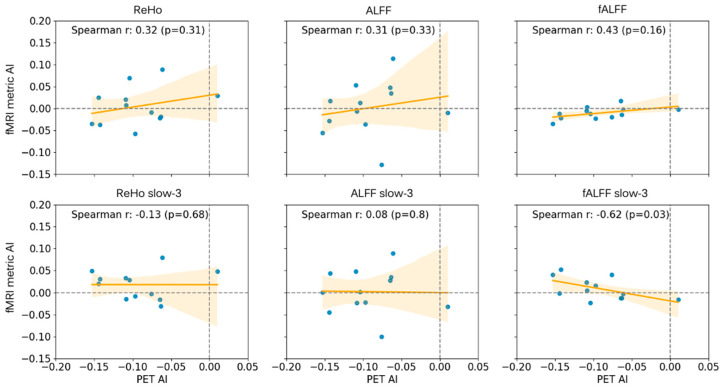
Correlation charts of PET and fMRI asymmetry indices with linear regression lines and the associated Spearman correlation coefficients. The horizontal axis shows the PET asymmetry index while the vertical axis shows the corresponding fMRI metric asymmetry index. AI: asymmetry index; PET: positron emission tomography; ReHo: regional homogeneity; ALFF: amplitude of low-frequency fluctuations; fALFF: fractional ALFF.

The asymmetry analysis showed a significantly negative AI for the PET (−0.09 ± 0.05 asymmetry index; *p* < 0.01; by design) and fALFF (−0.01 ± 0.01 asymmetry index; *p* < 0.05) (Figure 2A). Furthermore, a significant strong negative correlation was found between the PET and fALFF slow-3 (r = −0.62; *p* < 0.05) (Figure 2B, Figure 3 bottom-right). Additionally, significant correlations were found amongst the fMRI metrics, notably a strong negative correlation between fALFF and fALFF slow-3 (r = −0.58; *p* < 0.05) (Figure 2B). Following the asymmetry indices and the correlation trends (Figure 2 and Figure 3), the most successful at showing lateralization performance was PET (11/12—92%), followed by fALFF (10/12—83%) and ReHo slow-3 (7/12—58%).

## 4. Discussion

This study evaluated simultaneously acquired rs-fMRI metrics and cerebral glucose uptake in patients with drug-resistant TLE for lateralization of the EZ. Asymmetry indices of fALFF provided significant lateralization of the EZ in agreement with patterns of PET hypometabolism. Moreover, the asymmetry indices of FDG-PET and fALFF slow-3 demonstrated a significant negative correlation.

In most cases across the literature, hypometabolism often co-localizes with the irritative zone [35]; therefore, the FDG-PET has been established as a valuable indicator of the functionally abnormal areas, with often interictally reduced glucose metabolism [36]. In 2013, Donaire et al. identified in an EEG-fMRI study increased BOLD activity within the hypometabolic regions and showed high spatial correspondence between interictal epileptiform activity and the FDG-PET abnormal regions in a drug-resistant young (5–25 y) epilepsy cohort [35]. In 2015, Nugent et al. found positive correlations between glucose uptake and both ALFF and ReHo in a healthy cohort, but the correlation of ALFF and glucose uptake was significantly lower in a temporal lobe epilepsy cohort [37]. This indicates that the BOLD signal was altered under the epileptic condition. These findings indicate a positive correlation between BOLD activity and glucose uptake. Multiple studies reported on increased hippocampal ALFF and ReHo in mesial temporal lobe epilepsy cohorts [15], with Chen et al. in 2017 specifically indicating that increased ReHo might be a biomarker for the EZ [13]. This was supported by Wang et al. in 2020 and 2021, who reported that (1) ReHo showed the highest regional and voxel-wise correlations with glucose uptake in an MTLE-HS cohort [24] and (2) coupling between fALFF and glucose uptake could aid surgical planning. To further validate the utility of rs-fMRI, Tang et al. in 2021 studied the concordance between SEEG and rs-fMRI and found strong overlaps (in 73.7% of patients in a cohort with DRE) between ALFF and SEEG clusters, while ReHo showed unconvincing results [16].

The asymmetry indices results were as expected according to the previous literature for FDG-PET, with the presumed epileptogenic area showing lower values compared with the contralateral region [36]. Regarding the rs-fMRI metrics, fALFF showed a significant decrease in the hypometabolic region compared with the contralateral side. This is very interesting since there was a significant strong negative correlation between the fALFF slow-3 and FDG-PET indices. Hints of the negative correlation can be observed in Figure 2, where the fALFF from the slow-3 band shows lower values in the occipital area while all other metrics show high values in the same area.

Nevertheless, conflicting results can be found across the literature regarding rs-fMRI metrics in TLE patients. Reyes et al. found decreased fALFF across the temporal and frontal lobes but mainly in the ipsilateral amygdala and hippocampus in a cohort of TLE patients with mesial temporal sclerosis [38]. Similarly, a decreased fALFF was found in the ipsilateral hippocampus in a cohort of mesial TLE patients with hippocampal sclerosis [21]. Furthermore, Qiao and Niu, among others, reported significantly increased fALFF in bilateral thalami and reduced fALFF in the medial prefrontal lobes bilaterally in a cohort of complex partial seizure patients [39]. Additionally, reduced fALFF was found bilaterally in the middle frontal gyri in MR-negative TLE patients [40]. Yan et al. found decreased fALFF in the putamen and thalamus in childhood absence epilepsy patients [41]. These prior studies highlight the diversity of findings about the rs-fMRI metrics in temporal lobe epilepsy and warrant further studies to describe their value.

Since fALFF contains information about the amount of blood-oxygen-level-dependent activity, a physiological coupling between fALFF and FDG-PET is expected as higher demand for glucose should theoretically result in a higher fALFF, and vice versa. On the other hand, increased fALFF, while showing decreased glucose uptake, may indicate a compensatory mechanism for the neuronal activity. Since the 1990s, the focal glucose hypometabolism has been utilized as an indicator of the EZ, and its presence has been correlated with improved surgical outcomes [19]. It has also been observed that glucose hypometabolism does not necessarily correlate with neuronal loss [19]. In later years, the hypometabolic tendency in drug-resistant epilepsy has become associated with the inability of mitochondria to metabolize the incoming glucose and thus creating an energetical local disruption [42,43]. This speculation might explain why some studies find increased rs-fMRI metrics and why here we observe a strong negative correlation between the FDG-PET and fALFF slow-3. Furthermore, the filter in the slow-3 band (0.073–0.198 Hz) likely suppresses some of the activity and brings out the negative correlation between FDG-PET and fALFF slow-3 [44]. This might be especially important for TLE as FDG-PET hypometabolism in extra-temporal lobe epilepsy appears more sporadically [45,46].

The neurovascular coupling between the blood flow and the epileptogenic character should also be emphasized [47]. Correlations between cerebral blood flow (CBF) and fALFF have previously been established, showing increased fALFF related to increased CBF [48]. Abnormal neurovascular coupling and reduced CBF/fALFF ratio has been previously found in both early-onset and late-onset epilepsy with unknown etiology [49]. Therefore, increased fALFF, indicating higher regional CBF, makes sense as a mechanism to compensate for the increased neuronal activity given by the epileptogenic zone. This could also indicate further mechanisms such as vascular steal, i.e., a redistribution of blood flow, to compensate for the higher demand of the metabolically compromised tissue. Delving deeper into the frequency sub-bands of the BOLD spectrum could provide further evidence for epileptogenic markers, with the slow-3 band specifically capturing different physiological processes compared with the normal frequency range. However, to investigate this mechanism, separate vascular and neuronal contributions need to be studied and evaluated, for instance, by adding arterial spin labeling into the scanning protocol. Additionally, although patients were instructed not to sleep and were not anesthetized, the dimmed-light resting conditions and post-injection waiting period may have induced drowsiness, differentially affecting neurovascular coupling in epileptogenic versus healthy tissue. Lastly, the FDG-PET asymmetry indices showed a wider distribution compared with the fALFF. This indicates a higher sensitivity of the FDG-PET compared with rs-fMRI. Potentially, utilizing stronger magnetic fields could provide a boost in magnetic susceptibility and could therefore yield improved sensitivity of the rs-fMRI metrics [50].

The presented study was also subject to limitations. The clinical FDG-PET control database (63.8 ± 9.9 y) was considerably older than the TLE patient cohort (34.1 ± 13.1 y). While this database is incorporated into the medical software and is routinely utilized as a reference in clinical practice, the age mismatch could indeed potentially result in an overestimation of metabolic changes in younger patients. However, we emphasize that our primary analysis relied on within-patient asymmetry indices comparing ipsilateral to contralateral regions, which excludes any age effect. Furthermore, all 12 TLE patients showed concordant lateralization of the FDG-PET abnormality with prior EEG findings, including 2 patients with concordant SEEG findings. This strengthens the validity of FDG-PET as ground truth, particularly given the EEG and FDG-PET concordance. However, direct validation against surgical outcome or systematic SEEG data in larger cohorts remains necessary to establish the clinical utility of rs-fMRI metrics. Lastly, the clinical evaluation from nuclear medicine experts was based not only on the z-score maps, but mainly on the original images with the captured standard uptake values. Nevertheless, we encourage future studies to strive for age-matched normative databases.

Another concern was the low number of PET-positive patients. While our in-house clinical cohort extends beyond 12 patients, the presurgical workup does not always provide enough evidence for the temporal lobe nor a unilateral seizure onset. The inclusion criteria for the study were set to eliminate possible confounders due to inconclusive electrophysiological findings, and only 12 patients satisfied those criteria.

Additionally, to account for the distortions and dropout in the inferior temporal lobe areas due to air, the inferior temporal neocortex, surrounding white matter, and two inferior transverse slices were removed from the analysis upon visual assessment, to avoid an artificial decrease in the average rs-fMRI metrics. This systematic exclusion might bias the asymmetry indices towards zero and prevent the analysis of epileptogenic tissue. However, we believe this limitation also strengthens our findings rather than weakens them. The results show significant plausible relationships, despite a portion of the most relevant anatomy being excluded due to artifacts. This compromise reflects clinical practice, where clinical scans have priority over research scans. For future studies, we strongly encourage optimized fMRI scanning protocols with extended coverage and reduced sensitivity to B0 inhomogeneities, for instance, using higher spatial resolution, shorter echo times and/or multi-echo designs. Lastly, while the available sample size may limit statistical power, the detected differences, particularly for FDG-PET asymmetry indices—significant by design—suggest the chosen methodology was appropriate for the analysis. Nevertheless, our results should be interpreted cautiously, with future studies striving to replicate the results with larger unified cohorts and appropriate statistical corrections.

Our findings and the findings across the literature illustrate the complexity of the relationship between FDG-PET and rs-fMRI and strongly suggest that there may be numerous hidden factors contributing to hypometabolic activity. Future efforts using 7T MRI should ideally attempt to replicate the findings from 3T and provide theoretically stronger evidence toward disentangling the relationship between rs-fMRI and PET in epilepsy.

## 5. Conclusions

Our results demonstrate that the rs-fMRI metrics show potential to aid the lateralization of the EZ in TLE; however, we observed mixed results in terms of asymmetry indices. The fALFF seems to capture similar information as the FDG-PET. As this is a proof-of-principle study, the methodology would benefit from further applications with extended cohorts. Therefore, further research is encouraged, especially with an optimized 3T resting-state fMRI sequence and a dedicated control database, to uncover the exact rules of the FDG-PET and rs-fMRI coupling in temporal lobe epilepsy.

## Figures and Tables

**Table 1:** PET-MRI acquisition parameters. BOLD rs-fMRI: blood-oxygen-level-dependent resting-state functional MRI; T1w: T1-weighted; PET: positron emission tomography; MPRAGE: magnetization prepared rapid gradient echo; FDG: fluorodeoxyglucose; kg: kilogram; MBq: megabecquerel; EPI: echo planar imaging.

	PET	T1w	BOLD rs-fMRI
TR [ms]	-	2300	2340
TE [ms]	-	2.34	30
TI [ms]	-	900	-
Voxel size [mm]	2 × 2 × 2	0.5 × 0.5 × 0.5	3 × 3 × 3
Matrix size [voxels]	344 × 344 × 127	320 × 512 × 512	80 × 80 × 40
FDG dose [MBq/kg]	1.56 ± 0.13	-	-
Number of volumes	1	1	175
Sequence type	-	3D MPRAGE	2D EPI
Field strength [Tesla]	-	3	3
Flip angle [degrees]	-	8	90

**Table 2:** Patient demographics. FDG-PET. Sub encodes the {age}{sex}-{handedness}. M: male; F: female; R: right; L: left; FDG-PET: 18F-fluorodeoxyglucose positron emission tomography; SF: seizure frequency; d/w/m: day/week/month; y: yes; n: no; EH: epilepsy history; TCS: tonic–clonic seizures; BW: body weight; I-to-S delay: injection-to-scan delay; MBq: megabecquerel; EEG: electroencephalography; MEG: magnetoencephalography; SEEG: stereo-EEG; B: bilateral; T/P/F/O/I: temporal/parietal/frontal/occipital/insular; PS: perisylvian; W: widespread; Lat: lateralization; Loc: localization; n/a: not available.

Sub	SF	TCS	EH	FDG-PET Scan			Presurgical Workup					Outcome [Lat-Loc]
				Tracer Dose [MBq/kg]	BW [kg]	I-to-S Delay [min]	FDG-PET	EEG	MEG	MRI	SEEG	
59F-R	0.3/w	n	y	1.60	79	41	L T	L T	R T	N	L T; I	L T
46M-R	u	y	n	1.37	104	53	R T	B PS	n/a	N	B I	R T
30M-R	clusters	n	n	1.80	75	53	R T	R W	R T	N	R T; O	R T
51M-R	>1/d	y	n	1.55	75	54	R T; P	R T; P	R T; P	N	n/a	R T
25F-R	2.5/m	n	y	1.49	81	54	R T	R T	n/a	N	n/a	R T
16M-R	0.3/w	y	n	1.54	98	46	L T	L T; F	n/a	N	n/a	L T
34F-R	clusters	y	n	1.60	65	53	R T	W	W	N	n/a	R T
15M-R	>1/w	y	n	1.64	59	52	R T; P	B T; F	R P; I	N	n/a	R T
29F-R	5/m	y	n	1.49	51	47	R T; I; P	R W	R T	R T	n/a	R T
31F-R	1/w	y	n	1.46	55	55	R T	R T; F	R T	N	n/a	R T
35M-L	u	y	n	1.46	70	56	R T	R T; P; O	R T	B W	n/a	R T
38M-L	1/m	y	y	1.58	90	55	L T; F; O	L T; F	R T	R T	n/a	L T

## Data Availability

The datasets presented in this article are not readily available because of their clinical nature and sensitive information. Requests to access the datasets should be directed to the corresponding author.

## Abbreviations

The following abbreviations are used in this manuscript:

3T	3 Tesla
7T	7 Tesla
AI	Asymmetry index
AIs	Asymmetry indices
ALFF	Amplitude of low-frequency fluctuations
ASM	Antiseizure medication
BMMR	Biomedical Magnetic Resonance
BOLD	Blood-oxygen-level-dependent
BW	Body weight
CAPHRI	Care and Public Health Research Institute
CARIM	Cardiovascular Research Institute Maastricht
DRE	Drug-resistant epilepsy
EEG	Electroencephalography
EPI	Echo planar imaging
EZ	Epileptogenic zone
fALFF	Fractional amplitude of low-frequency fluctuations
FDG	Fluorodeoxyglucose
FDG-PET	18Fluorodeoxyglucose positron emission tomography
fMRI	Functional magnetic resonance imaging
FSL	FMRIB Software Library
FWHM	Full width at half maximum
HS	Hippocampal sclerosis
kg	Kilogram
MBq	Megabecquerel
MEG	Magnetoencephalography
MHeNs	Mental Health and Neuroscience Research Institute
MPRAGE	Magnetization prepared rapid gradient echo
MRI	Magnetic resonance imaging
MTLE	Mesial temporal lobe epilepsy
NUTRIM	Nutrition and Translational Research in Metabolism
ReHo	Regional homogeneity
rs-fMRI	Resting-state functional magnetic resonance imaging
SD	Standard deviation
SEEG	Stereo-electroencephalography
SUV	Standard uptake value
T1w	T1-weighted
TD	Total injected dose
TE	Echo time
TI	Inversion time
TLE	Temporal lobe epilepsy
TR	Repetition time
